# Social, demographic and behavioural determinants of SARS-CoV-2 infection: a case-control study carried out during mass community testing of asymptomatic individuals in South Wales, December 2020

**DOI:** 10.1017/S0950268822000620

**Published:** 2022-05-10

**Authors:** Daniel Rh. Thomas, Laia Homar Fina, James P. Adamson, Clare Sawyer, Angela Jones, Kelechi Nnoaham, Alicia Barrasa, A. Giri Shankar, Chris J. Williams

**Affiliations:** 1 Communicable Disease Surveillance Centre, Public Health Wales, Cardiff, Wales, UK; 2 UK Field Epidemiology Training Programme, UK Health security Agency, London, UK; 3 Executive Team, Cwm Taf Morgannwg University Health Board, Abercynon, Rhondda Cynon Taf, Wales, UK; 4 Health Protection Division, Public Health Wales, Cardiff, Wales, UK

**Keywords:** Case-control study, epidemiology, public health, SARS-CoV-2, transmission

## Abstract

Between 21 November and 22 December 2020, a SARS-CoV-2 community testing pilot took place in the South Wales Valleys. We conducted a case-control study in adults taking part in the pilot using an anonymous online questionnaire. Social, demographic and behavioural factors were compared in people with a positive lateral flow test (cases) and a sample of negatives (controls). A total of 199 cases and 2621 controls completed a questionnaire (response rates: 27.1 and 37.6% respectively). Following adjustment, cases were more likely to work in the hospitality sector (aOR 3.39, 95% CI 1.43–8.03), social care (aOR 2.63, 1.22–5.67) or healthcare (aOR 2.31, 1.29–4.13), live with someone self-isolating due to contact with a case (aOR 3.07, 2.03–4.62), visit a pub (aOR 2.87, 1.11–7.37) and smoke or vape (aOR 1.54, 1.02–2.32). In this community, and at this point in the epidemic, reducing transmission from a household contact who is self-isolating would have the biggest public health impact (population-attributable fraction: 0.2). As restrictions on social mixing are relaxed, hospitality venues will become of greater public health importance, and those working in this sector should be adequately protected. Smoking or vaping may be an important modifiable risk factor.

## Introduction

There is growing evidence that certain population groups are more likely to be affected by severe COVID-19. These include older people, males, pregnant women and people with pre-existing chronic disease or disability [1–4]. People in certain minority ethnic groups and those in public-facing occupations are also disproportionally affected [5–8], but this is a combination of the risks of acquisition and progression to severe disease.

A proportion of SARS-CoV-2 infections will present as asymptomatic or mild infections, particularly in younger people [9, 10], so studies of risk factors for acquiring infection based on those hospitalised will be biased. Compared to evidence on risks of severe infection, limited information is available on the social, demographic and behavioural factors associated with transmission of SARS-CoV-2 infection in the community. Information gathered through the Test, Trace, Protect programme focuses on forwards contact tracing rather than factors associated with acquisition of infection.

A pilot mass testing exercise was initiated in South Wales. Whole borough testing took place in Merthyr Tydfil (population approximately 60 000) [11] between 21 November and 20 December 2020, and was extended to lower Cynon Valley in Rhondda Cynon Taf County Borough Council (an area of about 25 000 population covering five electoral wards) from 5 to 22 December 2020. This was the second such initiative in the UK, after a pilot scheme in Liverpool [12], and the first in Wales. Testing was offered at community settings to asymptomatic people aged 11 and over living, working or studying in the two areas. Symptomatic people were asked to seek tests through other routes. A total of 47 619 lateral flow tests (LFTs) were carried out at 12 testing centres in Merthyr Tydfil and at eight testing centres in the Lower Cynon Valley. Of these, 1135 (2.4%) were positive. People taking part were older than those in the catchment areas, and more tests (55%) were carried out in women.

Rates of confirmed COVID-19 in this relatively deprived, former industrial area of the South Wales Valleys, have been consistently high [13]. This testing exercise presented an opportunity to conduct an epidemiological study to obtain information on factors associated with testing positive for SARS-CoV-2 in a high incidence setting, in order to inform the ongoing response.

## Methods

### Study design

Unmatched case-control. Target population was adults (18 years and over) living, working or studying in Merthyr Tydfil County Borough or electoral wards in the lower Cynon Valley, Rhondda Cynon Taf County Borough selected because they were areas of persistently high incidence. The study population was adults (18 years and over) attending community testing for at least one LFT. Cases were defined as all people attending community testing pilot receiving a positive LFT result. Controls were a sample of those with a negative LFT result.

### Recruitment of cases and controls

Data on the results of LFT were de-duplicated to provide the first LFT for each person. These data contained the test result and the mobile phone number which was provided on registration when attending for testing. Rolling recruitment was carried out during the mass testing period. We contacted all cases and for each case, we generated a random sample of 10 individuals from the list of individuals who were tested on the same day but had a negative test result (controls).

### Data collection

A questionnaire was designed in the software tool *Smart Survey* [14]. All newly tested individuals with a positive result (cases) and the sample of negatives (controls) were sent a SMS text message (see Supplementary Material 1) through the government portal texting service ‘*notify.gov*’ [15] asking them to complete an anonymous self-administered online questionnaire accessed via a hyperlink. To distinguish between cases and controls, a different link was sent to each group. We asked 37 questions on demographic and social factors, including age, ethnicity and occupation, area of residence, household structure, caring responsibilities and social interactions in the previous 10 days (see Supplementary Material 2).

### Analysis

Analysis was carried out using Stata v14 [16]. Response rates for cases and controls were calculated. The age distribution of cases responding was compared to all cases, and the age distribution of controls was compared with the sample selected for recruitment using Spearman rank test. We also compared controls to all people attending mass testing who had a negative result.

We constructed a directed acyclic graph to inform the analysis. Having symptoms was excluded from the multivariable analysis as this considered not to be in the causal pathway. Also, being in contact with a known COVID-19 case was excluded from multivariable analysis, as this would underlie all other associated factors.

Variables were grouped into four categories: (i) personal characteristics, (ii) occupational exposures, (iii) household exposures and (iv) social exposures. Unmatched univariate analysis was carried out using Stata v14 to identify social and demographic factors associated with testing positive. Odds ratios (OR) with 95% confidence intervals (CI) were calculated for each exposure variable using logistic regression. Small area deprivation status was assigned to cases and controls using their area of residence. Deprivation quintiles were calculated based on the distribution of Welsh Index of Multiple Deprivation [16] assigned to lower super output areas (LSOA) in Wales. Each participant was then classified into a deprivation quintile based on their LSOA of residence.

Multivariate analysis was then carried out by logistic regression to take account of potential confounders or effect modifiers, identified *a priori* or in the univariate analysis. First, all exposures were adjusted for all other exposures within each of the four exposure categories (i) to (iv). Those variables that remained significant at *P* < 0.05 were included in a further final multivariate analysis to identify those factors most important in predicting risk of infection, with a forward step-wise approach using Akaike information criterion as a comparative fit statistic. Due to collinearity between ‘place of work’ and ‘key worker’ fields, three new binary fields were created from the ‘key worker’ field: ‘health and social care worker’, ‘transport worker’ and ‘public service worker’.

Lastly, to assess the public health significance of the exposures identified through multivariable analysis, we calculated population-attributable fractions with 95% CI for those exposures that remained positively associated with testing positive after adjustment using *punafcc* post-estimation command in Stata [17]. In order to use the *punafcc* command it was necessary to recode the categorical ‘place of work’ field to create three new binary variables for ‘working in a healthcare setting’, ‘working in a social care setting’ and ‘working in a hospitality setting’. Other variables of interest were already in a binary format. Adjusted ORs were plotted against population-attributable fractions to investigate the relationship between personal risk and public health impact.

### Ethics approval

We carried out his study to inform the ongoing epidemic response; and as such this study was determined by Public Health Wales' Research and Development Office to be usual public health practice covered by Public Health Wales' Establishment Order, and covered by the COVID-19 privacy statement issued for the testing pilot [18]. Data were held and processed under Public Health Wales' information governance arrangements in compliance with the Data Protection Act, Caldicott Principles and Public Health Wales guidance on the release of small numbers. No data identifying protected characteristics of an individual were released outside Public Health Wales. Notify is a UK Government run platform which is a secure mass texting service. Notify is compliant with the Data Protection Act and any user data uploaded (e.g. phone numbers) are deleted after 7 days. Data which pass through the system are encrypted. Notify has been assessed and approved by the Cabinet Office Senior Information Risk Officer (SIRO). The SIRO checks this approval once a year. Notify is suitable for sending messages classified as ‘OFFICIAL’ or ‘OFFICIAL-SENSITIVE’ under the Government Security Classifications policy.

### Patient and public involvement statement

The study was set up rapidly, and neither patients nor the public were involved in the design and conduct of the case-control study. However, the questionnaire was piloted on local residents, and the study was closely linked to the evaluation of the whole area testing programme which included input from a wide range of stakeholders, including local community representatives. Headline findings from the study were shared with local residents by Cwm Taf Morgannwg University Health Board via media and social media [19].

## Results

### Response

SMS messages were sent to 735 positives and 6970 negatives aged 18 years or over and for whom we had a valid phone number for. There were a total of 4409 questionnaire attempts but only completed questionnaires were exported from the survey tool. A total of 199 cases and 2621 controls were recruited, giving response rates of 27.1 and 37.6% respectively.

Cases had a similar age distribution to all people testing positive during the pilot (Spearman's rank correlation, *P* = 0.07). Negative controls recruited had the same modal age group (50–59 years) as those selected to take part, but older people were over-represented in the control group (Spearman's rank correlation, *P* = 0.01). Negative controls had a similar age distribution to all people attending mass screening who tested negative (Spearman's rank correlation, *P* = 0.07).

### Symptoms

Nearly all (99.6%) of people attending the testing pilot reported being asymptomatic at the point of test registration. However, at the time of questionnaire completion, 87 of 198 (44%) cases taking part in the study reported symptoms compatible with COVID-19 (loss of sense of smell/taste, a new ongoing cough, or a fever) indicating that a proportion of those testing positive were pre-symptomatic.

### Factors associated with a positive LFT

Cases were more likely to be in younger age groups (Table 1). Only small numbers of cases (<10) and controls (81) classified themselves as being in an ethnic group other than white-British or Irish. Cases were slightly more likely to be in a White – other ethnicity (OR 1.23), but this was not statistically significant. The majority of cases and controls lived in areas classified as within the three most deprived quintiles. Cases were slightly more likely to live in the most deprived areas and slightly less likely to live in the least deprived areas but this effect did not reach statistical significance (Table 1).

**Table 1.** Personal characteristics in people testing positive for SARS-CoV-2 (cases) and controls, with odds ratios

		Cases		Controls		Univariate analysis			Multivariable analysis^1^		
		Exposed	%	Exposed	%	Odds ratio	95% CI	*P* value	Adjusted odds ratio	95% CI	*P* value
		*n* *=* 199		*n* *=* 2621							
**Age group**	18–20	4	2.0	46	1.8	2.77	0.88–8.70	0.081	1.38	0.35–5.35	0.644
	**21–29**	**26**	13.1	**215**	8.2	**3.85**	**2.00–7.42**	**0.000**	**2.54**	**1.17–5.50**	**0.018**
	**30–39**	**37**	18.6	**405**	15.5	**2.91**	**1.57–5.38**	**0.001**	1.75	0.84–3.68	0.137
	**40–49**	**47**	23.6	**507**	19.3	**2.95**	**1.63–5.35**	**0.000**	**2.02**	**1.00–4.08**	**0.050**
	**50–59**	**57**	28.6	**675**	25.8	**2.69**	**1.50–4.81**	**0.001**	1.87	0.95–3.70	0.072
	60–65	13	6.5	295	11.3	1.40	0.65–2.99	0.379	1.20	0.53–2.74	0.664
	Over 65	15	7.5	478	18.2	Ref.	–	–	Ref.	–	–
		*n* *=* 199		*n* *=* 2618							
Ethnicity	White British or Irish	192	96.5	2537	96.5	Ref.	–	–	Ref.	–	–
	White other	4	2.0	43	1.6	1.23	0.43–3.46	0.696	1.39	0.48–4.03	0.541
	Any other background	3	1.5	38	1.4	1.04	0.32–3.41	0.944	1.07	0.25–4.66	0.927
		*n* *=* 181		*n* *=* 2406							
Welsh deprivation quintiles	Most deprived	55	30.4	616	25.6	1.21	0.66–2.19	0.531	1.05	0.56–1.96	0.872
	2nd most deprived	65	35.9	823	34.2	1.07	0.60–1.91	0.823	0.96	0.53–1.77	0.906
	3rd most deprived	34	18.8	588	24.4	0.78	0.41–1.47	0.444	0.79	0.41–1.52	0.484
	4th most deprived	12	6.6	176	7.3	0.92	0.42–2.02	0.841	0.81	0.36–1.80	0.606
	Least deprived	15	8.3	203	8.4	Ref.	–	–	Ref.	–	–
		*n* *=* 179		*n* *=* 2380							
**Residence in catchment area**	**Yes**	**124**	**69.3**	**1864**	**78.3**	**0.62**	**0.45–0.87**	**0.005**	0.79	0.55–1.13	0.193
		*n* *=* 196		*n* *=* 2619							
**Smoke or vape**	**Yes**	**44**	**22.4**	**422**	**16.1**	**1.51**	**1.06–2.14**	**0.022**	**1.47**	**1.00–2.15**	**0.048**
**Place of work**		*n* *=* 193		*n* *=* 2504							
	Working from home or not currently working	51	26.4	1053	42.1	Ref.	–	–	Ref.	–	–
	Factory/industrial setting	13	6.7	174	6.9	1.54	0.82–2.90	0.177	1.38	0.70–2.74	0.350
	**Social care setting**	**11**	**5.7**	**74**	**3.0**	**3.07**	**1.53–6.14**	**0.002**	**2.60**	**1.25–5.39**	**0.010**
	Education	17	8.8	281	11.2	1.25	0.71–2.20	0.440	0.98	0.52–1.87	0.962
	**Healthcare setting**	**28**	**14.5**	**242**	**9.7**	**2.39**	**1.48–3.87**	**0.000**	**1.95**	**1.14–3.36**	**0.016**
	**Hospitality**	**11**	**5.7**	**42**	**1.7**	**5.41**	**2.63–11.12**	**0.000**	**4.93**	**2.29–10.60**	**0.000**
	Retail	5	2.6	113	4.5	0.91	0.36–2.34	0.850	0.81	0.31–2.12	0.664
	**Office setting**	**28**	**14.5**	**245**	**9.8**	**2.36**	**1.46–3.82**	**0.000**	**2.13**	**1.24–3.66**	**0.006**
	**Outside**	**6**	**3.1**	**50**	**2.0**	**2.48**	**1.02–6.05**	**0.046**	2.28	0.91–5.72	0.080
	**In prisons**	**1**	**0.5**	**1**	**0.0**	**20.65**	**1.27–334.82**	**0.033**	12.25	0.72–209.58	0.084
	In homes/businesses/premises you are not resident in	15	7.8	173	6.9	1.79	0.98–3.25	0.056	1.62	0.85–3.08	0.142
	Transport inc. deliveries	1	0.5	22	0.9	0.94	0.12–7.10	0.951	0.87	0.11–6.72	0.895
	**Other**	**6**	**3.1**	**34**	**1.4**	**3.64**	**1.46–9.07**	**0.005**	2.64	0.87–7.98	0.086
Key worker		*n* *=* 197		*n* *=* 2571							
	Not a key worker or not currently working	82	41.6	1269	49.4	Ref.	–	–	Ref.	–	–
	**Health and social care**	**45**	**22.8**	**406**	**15.8**	**1.72**	**1.17–2.51**	**0.005**	1.26	0.82–1.93	0.286
	Public safety	6	3.0	48	1.9	1.93	0.80–4.65	0.141	1.53	0.62–3.78	0.359
	Local and national government	10	5.1	207	8.1	0.75	0.38–1.47	0.397	0.52	0.25–1.08	0.080
	Education and childcare	19	9.6	321	12.5	0.91	0.55–1.53	0.738	0.62	0.35–1.11	0.107
	Food and necessary goods	12	6.1	113	4.4	1.64	0.87–3.10	0.126	1.22	0.63–2.36	0.554
	**Transport**	**8**	**4.1**	**46**	**1.8**	**2.69**	**1.23–5.89**	**0.013**	1.58	0.64–3.89	0.324
	Utilities, comms and financial services	11	5.6	145	5.6	1.17	0.61–2.25	0.630	0.78	0.38–1.62	0.504
	**Public service worker**	**4**	**2.0**	**16**	**0.6**	**3.87**	**1.26–11.83**	**0.018**	**3.59**	**1.12–11.51**	**0.032**

^1^Factors significantly associated with being a case in bold. Multivariable analysis adjusted for all other variables in table except ‘key worker’. Multivariable analysis of ‘key worker’ was carried out by adjusting for all variables in the tables except ‘place of work’.

Most cases and controls were resident within the catchment, but cases were less likely to be resident inside the catchment area (OR 0.62, 95% CI 0.45–0.87). Twenty-two per cent of cases reported smoking or vaping compared to 16% of controls (OR 1.51, 95% CI 1.06–2.14). Twenty-six per cent of cases (51/193) were either not working or were working from home, as compared to 42% of controls. Compared to those not currently working or working from home, cases were more likely to work in a social care setting (OR 3.07, 95% CI 1.53–6.14), in a healthcare setting (OR 2.39, 95% CI 1.48–3.87), in hospitality (OR 5.41, 95% CI 2.63–11.12), in an office (OR 2.36, 95% CI 1.48–3.82), in prison (OR 20.65, 95% CI 1.27–334.82), or in an ‘other’ setting (OR 3.64, 95% CI 1.46–9.07). In those who worked, cases were less likely to work from home (OR 0.43, 95% CI 0.52–0.73) (Table 2).

**Table 2.** Occupational exposures in people who reported that they work

		Cases			Controls			Univariate analysis			Multivariable analysis (*n* = 1912)		
		Total	Exposed	%	Total	Exposed	%	Odds ratio	(95% CI)	*P* value	Odds ratio	(95% CI)	*P* value
			*n* *=* 156			*n* *=* 1878							
**Working from home**	**Yes**	**156**	**16**	**10.3**	**1812**	**379**	**20.9**	**0.43**	**0.25–0.73**	**0.002**	**0.43**	**0.25–0.73**	**0.002**
			*n* *=* 159			*n* *=* 1803							
Work environment	Mostly outdoors	159	17	10.7	1803	179	9.9	1.09	0.64–1.84	0.758	1.02	0.60–1.73	0.949
			*n* *=* 169			*n* *=* 2063							
Travelled to work by public transport	Yes	169	7	4.1	2063	76	3.7	1.13	0.51–2.49	0.762	0.89	0.38–2.10	0.796
			*n* = 169			*n* *=* 2063							
Travelled to work by car share	Yes	169	8	4.7	2063	73	3.5	1.35	0.64–2.86	0.426	1.21	0.57–2.59	0.62

Factors significantly associated with being a case in bold. Multivariable analysis carried out by adjusting for all other variables in the table.

Univariate analysis of household exposures (Table 3) indicates that cases were more likely to live in larger households (OR for living with six or more people 4.43, 95% CI 1.79–10.95, using living alone as a reference), were more likely to live with a child aged under 11 years (OR 1.41, 95% CI 1.01–1.97), were more likely to live with someone aged 23–59 years (OR 1.60, 95% CI 1.16–2.19) and were more likely to live with a healthcare worker (OR 1.60, 95% CI 1.08–2.37). Cases were less likely to live with someone aged 60 years or over (OR 0.63, 95% CI 0.44–0.90) or live with someone working in education (OR 0.52, 95% CI 0.27–0.99).

**Table 3.** Household exposures in people testing positive for SARS-CoV-2 (cases) and controls, with odds ratios

		Cases		Controls		Univariate analysis			Multivariable analysis		
		Exposed	%	Exposed	%	Odds ratio	(95% CI)	*P* value	Odds ratio	(95% CI)	*P* value
		*n* *=* 192		*n* *=* 2558							
Type of residence	Private residence	185	96.4	2531	98.9	Ref.	–	–	Ref.	–	–
	Care facility or assisted living	1	0.5	6	0.2	2.28	0.27–19.04	0.447	2.31	0.25–21.67	0.463
	Student hall	1	0.5	2	0.1	6.84	0.62–75.79	0.117	5.42	0.41–72.38	0.201
	No fixed place	2	1.0	8	0.3	3.42	0.72–16.22	0.122	2.43	0.45–13.06	0.299
	**Other**	**3**	**1.6**	**11**	**0.4**	**3.73**	**1.03–13.49**	**0.045**	3.16	0.80–12.47	0.100
		*n* *=* 198		*n* *=* 2620							
Household size	Live alone	17	8.6	348	13.3	Ref.	–	–	Ref.	–	–
	1–2 others	95	48.0	1328	50.7	1.46	0.86–2.49	0.158	1.14	0.39–3.33	0.808
	**3–5 others**	**78**	**39.4**	**907**	**34.6**	**1.76**	**1.03–3.02**	**0.040**	1.02	0.32–3.26	0.969
	**6 or more**	**8**	**4.0**	**37**	**1.4**	**4.43**	**1.79–10.95**	**0.001**	2.05	0.48–8.78	0.334
		*n* *=* 199		*n* *=* 2628							
Live alone	Yes	13	6.5	273	10.4	0.60	0.34–1.07	0.085	0.79	0.25–2.49	0.682
**Live with someone <11 years**	**Yes**	**50**	**25.1**	**506**	**19.3**	**1.41**	**1.01–1.97**	**0.045**	1.35	0.85–2.15	0.210
Live with someone 11–17 years	Yes	41	20.6	453	17.2	1.25	0.87–1.78	0.229	1.20	0.75–1.93	0.441
Live with someone 18–22 years	Yes	33	16.6	392	14.9	1.13	0.77–1.67	0.526	0.98	0.61–1.58	0.946
**Live with someone 23–59 years**	**Yes**	**142**	**71.4**	**1601**	**60.9**	**1.60**	**1.16–2.19**	**0.004**	1.17	0.69–1.96	0.565
**Live with someone 60 + years**	**Yes**	**39**	**19.6**	**737**	**28.0**	**0.63**	**0.44–0.90**	**0.011**	0.65	0.38–1.11	0.112
		*n* *=* 199		*n* *=* 2628							
**Live with healthcare worker**	**Yes**	**33**	**16.6**	**290**	**11.0**	**1.60**	**1.08–2.37**	**0.019**	1.30	0.85–1.98	0.231
Live with care worker	Yes	4	2.0	53	2.0	1.00	0.36–2.78	0.995	0.73	0.25–2.15	0.571
Live with supermarket worker	Yes	13	6.5	107	4.1	1.65	0.91–2.98	0.100	1.49	0.80–2.78	0.205
**Live with education worker**	**Yes**	**10**	**5.0**	**243**	**9.2**	**0.52**	**0.27–0.99**	**0.048**	**0.44**	**0.22–0.86**	**0.016**
Live with children attending school	Yes	42	21.1	596	22.7	0.91	0.64–1.30	0.609	**0.58**	**0.35–0.95**	**0.031**
		*n* *=* 199		*n* *=* 2603							
Someone in household self-isolating	**Yes**	**57**	**28.6**	**221**	**8.5**	**4.32**	**3.09–6.06**	**0.000**	**3.92**	**2.73–5.62**	**0.000**

Factors significantly associated with being a case in bold. Multivariable analysis carried out by adjusting for all other variables in the table.

Although only a small number of respondents visited a pub in the preceding 10 days (8 cases, 38 controls), this was significantly associated with infection (OR 2.85) (Table 4). Cases were significantly less likely to have had household visitors, and were less likely to visit a shop or supermarket. Cases were not more likely to have caring responsibilities for someone outside their household. Cases were significantly less likely to have attended a face-to-face healthcare appointment in the preceding 10 days.

**Table 4.** Social contact in people testing positive for SARS-CoV-2 (cases) and controls, with odds ratios

		Cases		Controls		Univariate analysis			Multivariable analysis		
		Exposed	%	Exposed	%	Odds ratio	(95% CI)	*P* value	Odds ratio	(95% CI)	*P* value
		*n* *=* 199		*n* *=* 2610							
Caring responsibilities	Yes	39	19.6	649	24.9	0.74	0.51–1.06	0.097	0.66	0.42–1.05	0.080
		*n* *=* 198		*n* *=* 2598							
Attended an event of gathering	Yes	10	5.1	224	8.6	0.56	0.29–1.08	0.084	0.61	0.30–1.23	0.166
		***n* *=* 199**		***n* *=* 2628**							
**Household visitors in last 10 days**	**Yes**	**29**	**14.6**	**221**	**8.4**	**0.30**	**0.20–0.45**	**0.000**	**0.48**	**0.35–0.66**	**0.000**
		*n* *=* 198		*n* *=* 2611							
Household overnight visitors in last 10 days	Yes	11	5.6	176	6.7	0.81	0.43–1.52	0.520	2.09	0.95–4.59	0.067
		***n* *=* 199**		***n* *=* 2618**							
**Attended face-to-face healthcare appointment**	**Yes**	**13**	**6.5**	**355**	**13.6**	**0.45**	**0.25–0.79**	**0.006**	**0.48**	**0.25–0.91**	**0.026**
		***n* *=* 199**		***n* *=* 2622**							
**Visited a shop**	**Yes**	**158**	**79.4**	**2409**	**91.9**	**0.34**	**0.23–0.49**	**0.000**	**0.46**	**0.28–0.76**	**0.003**
		***n* *=* 199**		***n* *=* 2628**							
**Visited a supermarket**	**Yes**	**99**	**49.7**	**1830**	**69.6**	**0.43**	**0.32–0.57**	**0.000**	**0.52**	**0.36–0.76**	**0.001**
		***n* *=* 199**		***n* *=* 2628**							
**Visited a pub**	**Yes**	**8**	**4.0**	**38**	**1.4**	**2.85**	**1.31–6.21**	**0.008**	**4.54**	**1.82–11.32**	**0.001**
		*n* *=* 199		*n* *=* 2628							
Visited a restaurant or pub serving food	Yes	16	8.0	222	8.4	0.95	0.56–1.61	0.842	1.06	0.58–1.96	0.841
		*n* *=* 199		*n* *=* 2628							
Visited a gym/leisure centre	Yes	9	4.5	108	4.1	1.11	0.55–2.22	0.778	0.84	0.35–1.97	0.681
		*n* *=* 198		*n* *=* 2604							
Face to face conversation <2 m, >15 min with people outside household	No-one	85	42.9	1094	42.0	Ref.	–	–	Ref.	–	–
	1–9 people	88	44.4	1289	49.5	0.88	065–1.20	0.412	1.07	0.74–1.56	0.717
	10 or more people	25	12.6	221	8.5	1.46	0.91–2.33	0.116	**1.81**	**1.03–3.17**	**0.039**
		*n* *=* 198		*n* *=* 2615							
Wearing a mask when leaving home	None of the time	5	2.5	43	1.6	Ref.	–	–	Ref.	–	–
	Some of the time	48	24.2	702	26.8	0.59	0.22–1.55	0.284	0.94	0.25–3.55	0.923
	Most of the time	145	73.2	1870	71.5	0.67	0.26–1.71	0.399	0.84	0.22–3.18	0.803
		*n* *=* 172		*n* *=* 2228							
Wearing a mask when meeting others outside	Never	16	9.3	181	8.1	Ref.	–	–	Ref.	–	–
	Rarely or sometimes	30	17.4	515	23.1	0.66	0.35–1.24	0.194	**0.44**	**0.22–0.91**	**0.027**
	Usually or always	126	73.3	1532	68.8	0.93	0.54–1.60	0.794	0.68	0.34–1.35	0.268
		***n* *=* 176**		***n* *=* 2175**							
**Wearing a mask when meeting others inside**	**Never**	**3**	**1.7**	**130**	**6.0**	**Ref.**	–	–	**Ref.**	–	–
	**Rarely or sometimes**	**28**	**15.9**	**325**	**14.9**	**3.73**	**1.12–12.49**	**0.033**	**6.22**	**1.33–29.21**	**0.021**
	**Usually or always**	**145**	**82.4**	**1720**	**79.1**	**3.65**	**1.15–11.62**	**0.028**	**5.30**	**1.17–23.91**	**0.030**

Factors significantly associated with being a case in bold. Multivariable analysis carried out by adjusting for all other variables in the table.

Cases were more likely than controls to report having been in contact with someone who has been told that they have a positive COVID-19 test in the last 10 days (OR 2.23, 95% CI 1.63–3.05), and more likely to report someone in the household currently self-isolating because they had been in contact with someone with COVID-19 (OR 4.32, 95% CI 3.09–6.06). It is possible that if there was a delay in the case completing the questionnaire, the case may be reporting someone self-isolating due to contact with themselves, but our assumption is that this relates to contact with another confirmed case.

When asked about wearing face masks, most people (>70%) reported wearing a mask most of the time when leaving home. Cases reported being more likely to wear a face mask when meeting others inside. This remained significant after adjusting for all other social contact variables (Table 4).

**Fig. 1.**
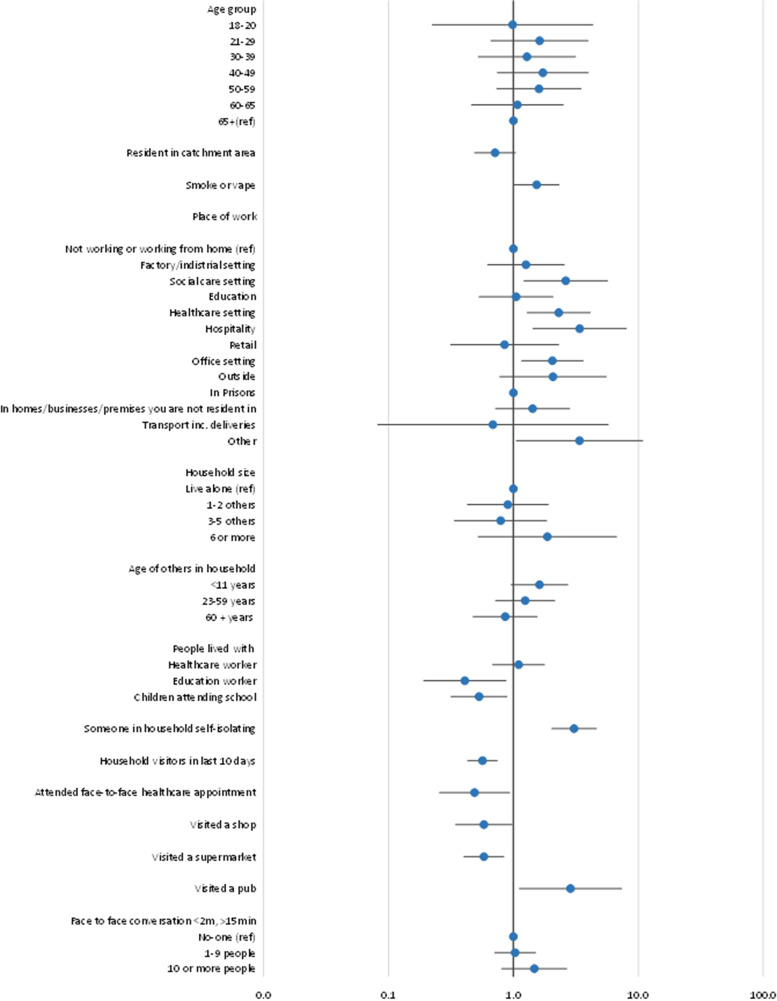
Final multivariable model: Forest plot showing adjusted odds ratios (aOR) for determinants of testing positive for SARS-CoV-2 in two areas of South Wales taking part in a community testing pilot, December 2020. aOR with 95% confidence intervals are given for those factors significant (*P* < 0.05) in univariate analysis. Odds ratios greater than one represent an increased risk; odds ratios less than one represent a decreased risk. 95% confidence intervals not crossing one reflect that the odds ratio is statistically significant.

The final multivariable model (Fig. 1) identified working in the hospitality sector (pubs, bars, restaurants, hotels, betting shops) (aOR 3.39, 95% CI 1.43–8.03), working in a social care setting (aOR 2.63, 95% CI 1.22–5.67), working in a healthcare setting (aOR 2.31, 95% CI 1.29–4.13), living with someone who is self-isolating (aOR 3.07, 95% CI 2.03–4.62), visiting a pub in the preceding 10 days (aOR 2.87, 95% CI 1.11–7.37) and smoking or vaping (aOR 1.54, 95% CI 1.02–2.32) as significant factors.

### Population-attributable fractions

**Fig. 2.**
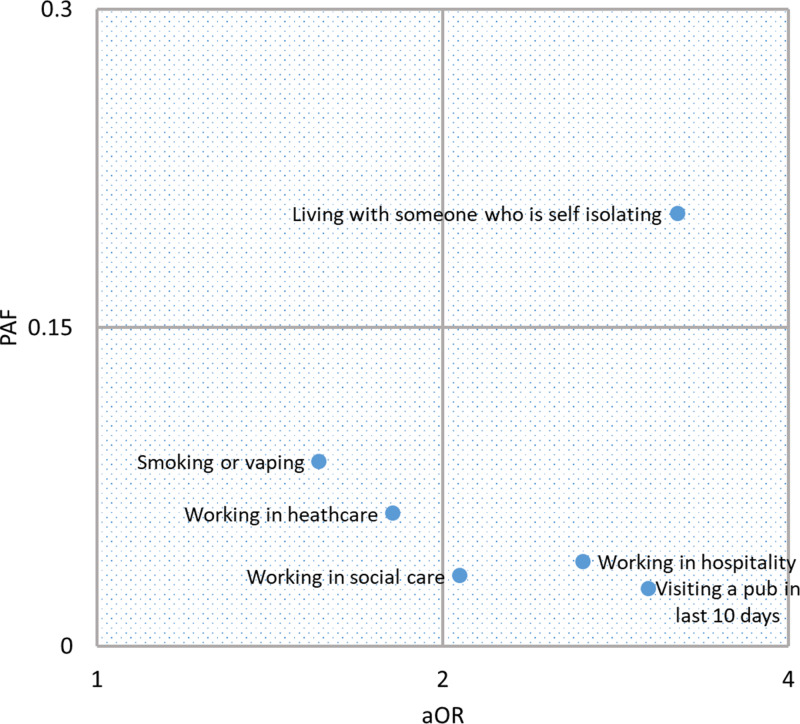
Relationship between personal risk, expressed as adjusted odds ratio (aOR) and public health impact expressed as population-attributable fraction (PAF) for exposures associated with testing positive during the SARS-CoV-2 mass testing pilot in Merthyr Tydfil and lower Cynon Valley, 21 November to 20 December 2020. aOR is plotted on a log scale.

Population-attributable fractions were 0.040 (95% CI 0.020–0.059) for working in the hospitality sector, 0.033 (95% CI 0.011–0.055) for working in a social care setting, 0.063 (95% CI 0.024–0.100) for working in a healthcare setting, 0.204 (95% CI 0.166–0.241) for living with someone who is self-isolating because they had been in contact with a confirmed case, 0.027 (95% CI 0.015–0.040) for visiting a pub in the preceding 10 days and 0.087 (95% CI 0.021–0.149) for smoking or vaping (Fig. 2). Adjusted ORs for the recoded binary variables ‘working in a healthcare setting’, ‘working in a social care setting’ and ‘working in a hospitality setting’ were reduced slightly to 1.81, 2.07 and 2.65, respectively, but remained significant. Plotting PAF against aOR provides an indication of the relationship between public health and personal risk, and how this might change with changes to interventions.

## Discussion

This study provides insight into the factors determining likelihood of testing positive for SARS-CoV-2. The study was carried out at the peak of the second wave of COVID-19 in the UK, and took place in localities which at the time had some of the highest rates of infection in the UK. We have demonstrated that it is possible to rapidly design and implement an epidemiological study to take place alongside a mass testing exercise, without compromising the primary objective of the exercise.

We investigated factors determining the likelihood of testing positive for SARS-CoV-2 in order to provide insight into transmission in the community. In this community, household exposures appeared to be important. Household mixing is largely hidden, and may be perceived as lower risk than mixing with people from outside the home [20]. Whilst media attention has focussed on adherence to restrictions affecting social contact outside the home, for example, travelling to exercise, attending work or going to school, transmission within households is being increasingly recognised as an important factor in the epidemiology of SARs-CoV-2 [21–23]. The former mining areas of the South Wales Valleys are characterised by close-knit communities, and have similarities with post-industrial towns in the North of England. One in five asymptomatic infections could have been prevented by avoiding contact with someone within the same household. Further work should be carried out to better understand the barriers to infection prevention and control within households, and how best to strengthen prevention and control advice, for example, using online tools [24, 25]. Further work could also be done to examine risks associated with household composition, whilst living with children may present an obvious risk, living with older people may increase protective behaviour.

Working in the hospitality sector, and visiting the pub were significant risks but at the time of this study were relatively infrequent exposures. The study took place before national ‘lockdown’ restrictions were introduced in Wales on 20 December, but were during a time when activity in the hospitality sector was restricted [26, 27]. As restrictions on social mixing are relaxed it is likely that exposure in hospitality venues will become of greater public health importance, and people working in this sector should be protected.

Smoking and vaping are potentially modifiable risk factors, and should be investigated further. Evidence for an association between smoking and COVID-19 has been mixed. Some researchers have suggested biological bases for an association. Others have suggested that it may relate to increased ‘hand to mouth’ contact [28, 29]. Smoking confounds other risk behaviours not measured in this study.

Of equal interest are the exposures that were not associated with infection. The policy to close schools and colleges has been debated, with concern that transmission risks are outweighed by the harms caused to children through lost education and socialisation [30]. We found no evidence that education settings provided a significant risk of transmission to adults: working in education, living with someone working in education or living with school age children were not associated with testing positive.

The safety of supermarkets, restaurant, gyms and leisure centre has also been debated [31]. Visiting these facilities did not appear to increase risk of infection. Of course, there is a great variety in the way people behave when shopping or socialising and variation in how shops and leisure facilities apply COVID-safe policies and procedures, and further work would be required to assess these hazards more fully.

Questions were asked about two specific non-pharmaceutical interventions: working from home and the wearing of face masks. Working from home was negatively associated with infection, and remains an important control measure. The results for mask wearing were unclear. In fact, in this study, people testing positive were more likely to report wearing a mask when meeting others inside. Qualitative methods could be used to investigate the behaviours associated with face mask use.

With so many associations investigated, it is always possible that some of our associations were chance findings. Moreover, statistically significant negative associations, such as living with an education worker, living with children who attend school, visiting a shop or supermarket and attending a face-to-face health appointment may be the result of confounding by an another unknown factor. For example, people attending a face-to-face health appointment may be more likely to be in a clinically vulnerable group and therefore may be mixing less. Selection of variables in the final multivariable model based on statistical significance may have limitations, as exposures only weakly associated in univariate analysis may assume greater importance when combined with other variables [32].

With a response rate of less than 40% it is possible that participants in our study were not representative of those people taking up the offer of testing. Moreover, it is likely that those accepting a test were not representative of people living in the catchment areas. Analysis by Cwm Taf Morgannwg University Health Board found that those taking up testing were older and were resident in less deprived areas of the catchment area.

The questionnaire was designed as a quick online questionnaire, taking participants around 5–10 min to complete, with participants recruited by SMS text message. The personal mobile phone number used to recruit was that given by participants at time of registering for testing at the community testing site, and the number which their lateral flow device test result was subsequently texted to. However, it is possible that some people were excluded from our survey as they did not have a valid mobile phone number of their own, or that their digital literacy level was not sufficient to use the link to our online questionnaire. Although digital tools offer many advantages over traditional paper questionnaires, they do have limitations. Our survey tool only included fully completed questionnaires, whilst quite a large number of questionnaires were partially completed, as the questionnaire was anonymous it is not clear whether these represented failed attempts in people who went on to complete on a second attempt. Also, not all populations have similar access to and expertise in using smart phone technology, and this should be considered when interpreting these types of surveys.

All exposures were self-reported. Although this was an anonymous study, all responses to questions about behaviour may be subject to social desirability bias, and should be interpreted with caution.

As an oversight, we did not include ‘gender’ on our questionnaire, preventing us from investigating the role of gender in our analysis. Another possible limitation in this study is choice of outcome measure, LFT positivity. LFT is considered to be specific but not particularly sensitive [33–35]. There will be some misclassification of cases and controls, but given the prevalence of SARS-CoV-2 in this setting, this is not considered to have had any significant impact on the findings.

The power of the case-control study was restricted by the number of lateral flow device positives, the frequency of certain determinants (e.g. there were only two people in our study reporting working in a prison setting) and our response rate. Factors such as working in a prison whilst no longer significant after adjusting for other variables would warrant further investigation in future studies.

We used population-attributable fractions to assess where interventions could be most effective. However, one should consider this was a very specific population which may not have exposures representative of the general population; and the response rates were different between cases and controls. In addition, population-attributable fractions should be interpreted with caution as these assume all confounding has been controlled for and a causal association between the exposure and the outcome.

Mass testing as a control measure has proved controversial [36, 37], but where it is undertaken, associated epidemiological studies can add to the knowledge about transmission risks. Combining this with the calculation of attributable fractions helps to focus on the major drivers of transmission, in order to produce evidence-based responses.

## Data Availability

The data that support the findings of this study are available on request from Public Health Wales. However to ensure compliance with information governance arrangements, certain restrictions may apply.

## Supplementary material

For supplementary material accompanying this paper visit https://doi.org/10.1017/S0950268822000620. click here to view supplementary material
