# Supplementary material for: Social, demographic and behavioural determinants of SARS-CoV-2 infection: a case-control study carried out during mass community testing of asymptomatic individuals in South Wales, December 2020
Source: Epidemiol Infect. 2022 May 10;150:e115. doi: 10.1017/S0950268822000620 (PMC9203360; doi:10.1017/S0950268822000620)
Supplement: Supplementary file 1 [file S0950268822000620sup001.zip › Merthyr_case_control__paper_2a_supp2.pdf]

Supplementary Material 2: Questionnaire

Merthyr Tydfil - Mass Testing questionnaire

1. Introduction

Please help us investigate why rates of Covid infection are high in your area.

If you are 18 or over, please fill in this ANONYMOUS short questionnaire to help Public Health Wales understand how we can better help fight the pandemic.

This will take approximately 10 minutes of your time. Taking part is VOLUNTARY.

It is important that you answer honestly. We will not link this questionnaire with your personal details (phone number, date of birth etc) in any way.

We will keep the data we collect safe, and use the information to understand risk factors for infection. This will help us control the pandemic.

2. Site

1. Where are you having a screening test today?

- ☐ Aberfan Community Centre
- ☐ Alpha House, Heolgerrig, CF48 1RP
- ☐ Bedlinog RFC, The Square, Bedlinog, CF46 6TG
- ☐ Clinic, Chestnut Way, Gurnos, CF47 9SB
- ☐ Cefn Community Centre, Old Drill Hall, Cefn Coed, CF48 2NA
- ☐ Dowlais Community Centre, Station Road, Dowlais, CF48 3LW
- ☐ Dowlais Engine House, High Street, Dowlais, CF48 3TB

- [ ] Merthyr Tydfil Leisure Centre
- [ ] Pontsticill Memorial Hall, Vaynor Road, Pontsticill CF48 2TU

3. COVID-19 Exposures

This section will cover information about your exposure to COVID-19

2. Are you currently experiencing a loss of sense of smell/taste, a new ongoing cough, or a fever?

- [ ] Yes
- [ ] No

3. In the last 10 days, have you had contact with someone who has been told that they have a positive test result for COVID-19?

- [ ] Yes
- [ ] No

4. Is this the first screening event for COVID-19 you have attended (which is not at your work place)?

- [ ] Yes
- [ ] No

4. Personal Information

This section will collect information about where you but **will not be linked to any other records**

5. Please select your age group

- [ ] 18-20

- [ ] 21-29
- [ ] 30-39
- [ ] 40-49
- [ ] 50-59
- [ ] 60-65
- [ ] 65+

6. Please select which ethnic group you identify as:

- **White**

- [ ] British
- [ ] Irish
- [ ] Other

- **Asian or Asian British**

- [ ] Indian
- [ ] Pakistani
- [ ] Bangladeshi
- [ ] Any other Asian background

- **Mixed**

- [ ] White and Black Caribbean
- [ ] White and black African
- [ ] White and Asian
- [ ] Any other mixed background

- **Black or Black British**

- [ ] Caribbean
- [ ] African
- [ ] Any other black background

- **Other Ethnic Group**

- [ ] Chinese
- [ ] Any other Ethnic Group
- [ ] I do not wish to disclose my ethnic origin

7. Please provide the postcode of the address you currently live at (for more than 3 nights per week):

8. Do you smoke cigarettes/vape?

- [ ] Yes
- [ ] No

5. Your occupation

This section covers how and where you work

9. Are you classed as a "key worker" by the Welsh Government? If yes, please select which group:

- [ ] Yes - Health and social care workers (e.g. doctors, nurses, social worker, NHS administrative workers)
- [ ] Yes - Public safety (emergency workers) and national security workers (e.g. fire & rescue, police staff)
- [ ] Yes - Local and national government workers (e.g. civil servants, council workers, charity sector staff)

- [ ] Yes - Education and childcare workers (e.g. childminders, teachers, cleaners and caterers in schools and colleges)
- [ ] Yes - Food and other necessary goods workers (e.g. food factory workers, farmers, food delivery drivers)
- [ ] Yes - Transport workers (e.g. bus drivers, train drivers, haulage drivers, warehousing and distribution workers)
- [ ] Yes - Utilities, communication and financial services workers (gas works, electricians, builders, IT staff, bank staff)
- [ ] Yes - Key public service workers (e.g. hotel workers, prison workers, funeral home workers)
- [ ] I am not currently working
- [ ] No - I am not a key worker

10. Do you work mostly indoors or outdoors?

- [ ] Mostly indoors
- [ ] Mostly outdoors
- [ ] I am not currently working

11. Are you currently working from home?

- [ ] Yes
- [ ] No
- [ ] Split between work place and home
- [ ] I am not currently working

6. Occupation - details

12. How did you travel to work in the last 10 days (tick all that apply)?

- [ ] Private car / Motorbike
- [ ] Car share
- [ ] Taxi
- [ ] Bus
- [ ] Train
- [ ] On foot
- [ ] Bicycle

13. Please select the option which best describes where you work (physical location):

- [ ] Office based
- [ ] Healthcare facility based (including receptions, offices, clinical)
- [ ] Care facility based (care home, nursing home, assisted living facilities)
- [ ] Educational setting based (school, school office)
- [ ] In a prison
- [ ] In retail (supermarket, clothing shop)
- [ ] In hospitality (bar, restaurant, pub, betting shop, hotels)
- [ ] A factory/Industrial setting
- [ ] In homes/businesses/premises which you are not resident in
- [ ] Outside (in parks, farms or in the community)

☐ Other (please specify):

7. Your household

This section is asking about your household

14. Please select the size of your household (people who live in your house more than 3 nights per week):

☐ I live alone

☐ 1 - 2 other people

☐ 3 - 5 other people

☐ 6 or more people

15. Please select the age groups of the people who live in your house (please tick all which apply):

☐ Under 11 years old

☐ 11 - 17 years old

☐ 18 - 22 years old

☐ 23 - 59 years old

☐ 60 - 69 years old

☐ 70 + years old

☐ I live alone

16. Do you have any household members who are:(if none, please leave unticked):

☐ Healthcare workers

- [ ] Care home workers
- [ ] Supermarket workers
- [ ] Educational staff
- [ ] Children attending school

17. Which of the following best describes where you live (spend more than 3 nights a week):

- [ ] Private residence (including shared house/flat)
- [ ] A care home/assisted living facility
- [ ] Student halls of residence
- [ ] No fixed place of residence
- [ ] Other (please specify):

18. Is anyone in your household currently self isolating due to contact with someone who has COVID-19?

- [ ] Yes
- [ ] No

19. Has anyone who doesn't live in your household spent the night at your house in the last 10 days?

- [ ] Yes
- [ ] No

20. Has anyone who doesn't live in your household visited you inside your home for more than 15 minutes, in the last 10 days? *

- [ ] Yes
- [ ] No

8. Household visitor details

21. How often have you had visitors from outside your household, inside your house for more than 15 minutes, in the last 10 days?

- [ ] Once
- [ ] Twice
- [ ] Three or more times

22. Please describe the relationship you have to the person/people who visited (you can select more than one response):

- [ ] Family
- [ ] Friends
- [ ] Household maintenance (cleaner, boiler check etc)
- [ ] Childcare provision
- [ ] Sexual partner
- [ ] Other (please specify):

23. Was alcohol consumed any of the times when someone who does not live with you, visited your home for more than 15 minutes?

- [ ] Yes - by me
- [ ] Yes - by others

- [ ] Yes - by me and others
- [ ] No

9. Caring responsibilities

24. Have you provided care for someone outside of your household in the last 10 days?(e.g. elderly individuals, disabled individuals, young children)

- [ ] Yes
- [ ] No

10. Events and Gatherings

25. Have you attended an event or a gathering with people you don't live with, in the last 10 days?

- [ ] Yes
- [ ] No

11. Event and gathering details

26. Roughly how many people were at the event(s)/gathering(s) you attended in the last 10 days

- [ ] 1-5
- [ ] 6-10
- [ ] 11-15
- [ ] 15+

27. What kind of gathering/event have you attended in the last 10 days?

- [ ] Gathering/event inside a venue/structure (pub, bar, restaurant)
- [ ] Gathering/event inside a friend's/family member's home
- [ ] Gathering/event inside a stranger's home
- [ ] Gathering/event inside my home
- [ ] Gathering/event in an outdoor setting
- [ ] Wedding
- [ ] Funeral/Wake
- [ ] Other (please specify):

28. Who did you attend the event(s)/gathering(s) with in the last 10 days?

- [ ] People from within your household
- [ ] People from outside your household
- [ ] Strangers
- [ ] Other (please specify):

29. Was there alcohol consumed at the event you attended in the last 10 days?

- [ ] Yes - by myself
- [ ] Yes - by others
- [ ] Yes - by myself and others
- [ ] No

12. Your Behaviours

30. Have you attended a health care appointment, face to face, with a medical practitioner (e.g. doctor, nurse, dentist) in the last 10 days?

- [ ] Yes
- [ ] No

31. How many times have you visited a shop (supermarket, clothing retailer, newsagent etc) in the last 10 days?

- [ ] Not at all
- [ ] 1 - 3 times
- [ ] More than 3 times

32. Have you visited any of the following in the last 10 days (please select all which apply)?

- [ ] A pub
- [ ] A restaurant (including pubs which serve food)
- [ ] A gym / leisure center
- [ ] A supermarket
- [ ] Other (please specify):

33. Have you travelled outside of Merthyr Tydfil Borough in the last 10 days? Please select all which apply

- [ ] Yes - to a neighboring local authority (e.g. Rhonnda Cynon Taf)
- [ ] Yes - to another part of Wales (e.g. Cardiff, Wrexham)

- [ ] Yes - to another country within the UK (England, Scotland, Northern Ireland)
- [ ] Yes - outside of the UK
- [ ] No

If YES please provide details of where you visited

34. How many people from outside of your household have you had a face to face conversation with (within 2 meters) in the last 10 days, for longer than 15 minutes (roughly)?

- [ ] 0 people
- [ ] Under 10 people
- [ ] 10 or more people

35. When you leave your home, do you wear a mask/face covering:

- [ ] Most of the time
- [ ] Some of the time - e.g. when indoors
- [ ] None of the time

36. When meeting people you do not live with, outdoors do you wear a mask/face covering:

- [ ] Always
- [ ] Usually
- [ ] Sometimes

- [ ] Rarely
- [ ] Never
- [ ] I do not meet friends or family outdoors

37. Thinking about people who you do not live with, when you meet them indoors (at work or socially), do you wear a mask/face covering?

- [ ] Always
- [ ] Usually
- [ ] Sometimes
- [ ] Rarely
- [ ] Never
- [ ] I do not meet any from outside my household inside
