# Supplementary material for: Social, demographic and behavioural determinants of SARS-CoV-2 infection: a case-control study carried out during mass community testing of asymptomatic individuals in South Wales, December 2020
Source: Epidemiol Infect. 2022 May 10;150:e115. doi: 10.1017/S0950268822000620 (PMC9203360; doi:10.1017/S0950268822000620)
Supplement: Supplementary file 1 [file S0950268822000620sup001.zip › Merthyr_case_control__paper_2a_supp1.pdf]

14:10

NHS Wales

Text Message
Today 10:21

Public Health Wales NHS Trust: Hello, you recently attended a mass screening event for coronavirus in Merthyr Tydfil Borough or in the Lower Cynon Valley area. Public Health Wales is investigating reasons for high numbers of coronavirus cases in the area. We would be grateful if you would complete an anonymous 10 minute questionnaire about your movements in the 10 days before your test, EVEN IF YOU TESTED NEGATIVE, to help us understand how the disease is spreading: https://www.smartsurvey.co.uk/s/5QR4F6/

Text Message
